# The Changes of Expression and Methylation of Genes Involved in Oxidative Stress in Course of Chronic Mild Stress and Antidepressant Therapy with Agomelatine

**DOI:** 10.3390/genes11060644

**Published:** 2020-06-11

**Authors:** Paulina Wigner, Ewelina Synowiec, Paweł Jóźwiak, Piotr Czarny, Michał Bijak, Gabriela Barszczewska, Katarzyna Białek, Janusz Szemraj, Piotr Gruca, Mariusz Papp, Tomasz Śliwiński

**Affiliations:** 1 Laboratory of Medical Genetics, Faculty of Biology and Environmental Protection, University of Lodz, 90-236 Lodz, Poland; paulina.wigner@gmail.com (P.W.); ewelina.synowiec@biol.uni.lodz.pl (E.S.); gabriela.barszczewska@unilodz.eu (G.B.); biaalek.k@gmail.com (K.B.); 2 Department of Cytobiochemistry, Faculty of Biology and Environmental Protection, University of Lodz, 90-236 Lodz, Poland; pawel.jozwiak@biol.uni.lodz.pl; 3 Department of Medical Biochemistry, Medical University of Lodz, 92-216 Lodz, Poland; piotr.czarny@umed.lodz.pl (P.C.); janusz.szemraj@umed.lodz.pl (J.S.); 4 Department of General Biochemistry, Faculty of Biology and Environmental Protection, University of Lodz, 90-236 Lodz, Poland; michal.bijak@biol.uni.lodz.pl; 5 Polish Academy of Sciences, Institute of Pharmacology, 31-343 Krakow, Poland; gruca@if-pan.krakow.pl (P.G.); nfpapp@cyfronet.pl (M.P.)

**Keywords:** chronic mild stress model, agomelatine, oxidative stress, gene expression and methylation

## Abstract

Preclinical studies conducted so far suggest that oxidative stress processes may be associated with the mechanism of depression development. This study shows the effects of chronic administration of agomelatine on expression and the methylation status of *Sod1*, *Sod2*, *Gpx1*, *Gpx4*, *Cat*, *Nos1,* and *Nos2* in the brain stricture and blood in the chronic mild stress (CMS) animal model of depression. The animals were exposed to the CMS procedure and treatment with agomelatine (10 mg/kg/day, IP) for five weeks and then were sacrificed. TaqMan Gene Expression Assay, Western blot, and methylation-sensitive high-resolution melting techniques were used to evaluate mRNA and protein expression of the genes, and the methylation status of their promoters. *Gpx1*, *Gpx4,* and *Sod2* expression in the PBMCs and *Sod1* and *Sod2* expression in the brain were reduced in the stressed group after agomelatine administration. CMS caused an increase in the methylation of the third *Gpx4* promoter in peripheral blood mononuclear cells and *Gpx1* promoter in the cerebral cortex. Additionally, stressed rats treated with agomelatine displayed a significantly lower Gpx4 level in the hypothalamus. The results confirm the hypothesis that the CMS procedure and agomelatine administration change the expression level and methylation status of the promoter region of genes involved in oxidative and nitrosative stress.

## 1. Introduction

Depression is a common and serious mental disorder. According to a report by the World Health Organization, 350 million people suffer from depression globally. Additionally, 800,000 people in the world commit suicide every year [1,2]. Depression is a multifactorial disease with a complex development mechanism. Previous studies have shown that antioxidant disturbances play an important role in the pathogenesis of neurological and psychiatric diseases, particularly depression [3]. This may be due to the fact that brain has a lower antioxidant capacity than other organs and thus is more sensitive to oxidative disorders [3]. More specific, the reduced levels of enzymatic and non-enzymatic antioxidants, including glutathione, zinc, uric acid, vitamins A, E, C, coenzyme Q10, glutathione peroxidase (GPx), superoxide dismutase (SOD), and catalase (CAT) were observed in the course of depression. Moreover, the low activity of GPx and SOD may be associated with severity of the disease. However, in the case of the enzymes, the obtained results are contradictory [4,5,6,7,8,9,10,11,12,13,14,15,16,17]. Additionally, a post-mortem study had shown an increased level of prooxidative enzyme, xanthine oxidase, in the serum and thalamus [18,19]. The overproduction of the reactive oxygen species observed in the course of depression may lead to damage to the important biomolecules. Thus, patients with depression were characterized by an elevated level of markers of peroxidation damage to lipid and DNA, including 8-F2-isoprostane (8-iso-PGF2α), malondialdehyde (MDA), and 8-hydroxy-2-deoxyguanosine [4,5,6,7,8,9,10,11,12,13,14,15,16,17]. Unfortunately, an excessive oxidative stress and poor antioxidant defense may lead to the induction of neuronal damage. The overproduction of reactive oxygen species may lead to the induction of apoptosis of neural cells [20]. Thus, patients with depression were characterized by a reduced volume of the hippocampus [21].

Studying the antioxidant properties of antidepressants is a worthwhile approach and can result in the development of promising new antidepressive drugs [22]. This is of particular importance, as one-third of patients do not respond to antidepressant treatment at all [23]. Oxidative stress intensification, which is observed in the course of depression, may influence the therapeutic action and side effects of antidepressants [24]. However, the mechanism of drug metabolism and action is not fully clear. Agomelatine is a melatonin analogue and a potentially strong antioxidant [25]. Melatonin is produced by the pineal gland and regulates the circadian rhythm as well as wakefulness and sleep by the duration of nocturnal secretion [26]. It plays an important role in the central nervous system as agonist at the melatonergic MT1 and MT2 receptors and as antagonist of the 5-HT2c receptors [27,28]. In addition to the MT1 and MT2 receptors, agomelatine is characterized by low affinity with other receptors, including adrenoceptors, dopamine, GABA (gamma-aminobutyric acid), muscarinic, histamine, benzodiazepine, and ion channels [29]. Therefore, agomelatine may regulate sleep disorders in patients suffering from depression and reduce severity of the disease [30]. However, the previous studies showed that agomelatine therapy is the most effective for patients with seasonal affective disorder, including seasonal depression. Animal and human studies confirmed that agomelatine showed beneficial effects on the regulation of disturbances of sleep and circadian rhythms [31,32,33,34]. Previously conducted studies confirmed the antioxidant nature of agomelatine in many different cells, although the results are inconsistent [35,36,37]. Therefore, the aim of the study presented herein was to investigate the effect of chronic mild stress (CMS; animal model of depression) and treatment with agomelatine in rats on mRNA and protein expression, and the promoter methylation of genes involved in oxidative stress. All of the genes analyzed in our study are characterized in Table S1.

## 2. Materials and Methods

### 2.1. Animals

Male Wistar Han rats (Charles River, Germany), weighing approximately 200–220 g, were housed as previously described by Wigner et al. (2020) [38]. All tests in the study were approved by the Bioethical Committee at the Institute of Pharmacology of the Polish Academy of Sciences in Krakow (Poland) and were conducted in compliance with the rules and principles of the 86/609/EEC Directive.

### 2.2. Chronic Mild Stress Procedure

The CMS experiment was conducted according to the method previously described [39]. The CMS procedure has been used because it is a well-validated and widely used animal model of depression, and therefore, much of the current understanding of the mechanisms of antidepressant drug action has been derived using this model.

First, the studied rats (30 animals) were apprenticed to for the weekly sucrose-intake test, which was carried out lasted 1 h following 14 h deprivation of water and food. Subsequently, the animals were divided into two groups matched for their sucrose intakes—control and stressed animals—as described by Wigner et al. (2020) [38]. Then, control and stressed animals were divided into subgroups based on their sucrose consumption values. Each subgroup (n = 6) received a vehicle (daily 10 mL/kg, IP) or agomelatine (daily 10 mg/kg, IP) for five weeks. The dose was selected on the basis of data in the literature and our own previous studies showing its efficacy in the CMS model of depression [40]. Then, weekly sucrose-intake tests were performed 24 h after the last dose. Finally, 24 h after the last sucrose intake test, the studied animals were decapitated, and samples of brain structures and blood were collected. Anesthesia was not used before decapitation.

### 2.3. Specimen Collection of Peripheral Blood Mononuclear Cells and Homogenates of Animal Brain Tissue

After decapitation of the studied animals, samples of blood were collected into vacutainer tube containing anticoagulants. Then, Gradisol L (Aqua-Med, Lodz, Poland) and differential migration of cells during centrifugation (400× *g*, 30 min, 4 °C) was used to isolate peripheral blood mononuclear cells (PBMCs). After centrifugation (400× *g*, 30 min, 4 °C), a pellet of PBMCs was stored at −20 °C until required.

Additionally, after removal, the studied animal brain structures were placed in liquid nitrogen and stored at −80 °C until required. Then, they were manually homogenized by FastGene^®^ Tissue Grinder (Nippon Genetics Europe, Düren, Germany) and sonicated. Finally, the prepared specimens were used to isolate DNA and RNA.

### 2.4. RNA Isolation, cDNA Synthesis, and Real-Time Analysis of the Expression of Genes Encoding Antioxidant Enzymes

Total RNA was isolated from PBMCs and frozen brain structures using the commercial spin column methods (GenElute Mammalian Total RNA Miniprep Kit, Sigma-Aldrich, Saint Louis, MO, USA; ISOLATE II RNA/DNA/Protein Kit, Bioline, London, UK, respectively) according to the manufacturer’s instructions. Then, spectrophotometric verification was used to measure the concentration and quality of the RNA samples, which were stored at −20 °C until use. The reaction of reverse transcription was performed using a High-Capacity cDNA Reverse Transcription Kit (Applied Biosystems, Foster City, CA, USA) according to the manufacturer’s protocol. The expression levels of all studied genes were detected and identified by real-time PCR using a TaqMan Universal Master Mix, no UNG, and species-specific TaqMan Gene Expression Assay—assay ID: Rn00566938_m1 (*Sod1*), Rn00690588_g1 (*Sod2*), Rn00577994_g1 (*Gpx1*), Rn00820818_g1 (*Gpx4*), Rn00560930_m1 (*Cat*), Rn00583793_m1 (*Nos1*), and Rn00561646_m1 (*Nos2*) (Thermo Fisher Scientific, Waltham, Massachusetts, USA). However, before starting the real-time PCR analysis, the authors carried out a review of the available literature and selected the 18S (*18S ribosomal RNA)* gene as the best reference gene. The reaction mixture consisted of total cDNA samples, TaqMan Universal Master Mix, no UNG (Applied Biosystems, Foster City, CA, USA), TaqMan Probe (Thermo Fisher Scientific, Waltham, MA, USA), and RNase-free water. The conditions of PCR included 10 min at 95 °C (enzyme activation), followed by 60 cycles of 30 s at 95 °C (denaturation) and 1 min at 60 °C (for annealing/extension). Real-time PCR runs were performed using a CFX96TM Real-Time PCR Detection System Thermal Cycler (Bio Rad Laboratories Inc., Hercules, CA, USA). The experiments were performed in duplicate for each sample. Gene expression was calculated in relation to that of the reference gene (ΔCt sample = Ct target gene—Ct reference gene). Next, the relative mRNA expression were calculated as fold = 2^−ΔCt^ sample. Moreover, the fold change in expression caused by antidepressant administration was assessed using the 2^−ΔΔCt^ method [41].

### 2.5. DNA Isolation, Methylation, and HRM Analysis

Total DNA was extracted from PBMCs and frozen brain structures by using commercial spin column methods (QIAamp DNA Mini Kit, Qiagen, Hilden, Germany; ISOLATE II RNA/DNA/Protein Kit, Bioline, respectively) following the manufacturer’s instructions. Then, spectrophotometric verification used for the measurement of the total concentration and quality of the isolated RNA samples, which were stored at −20 °C until use. Methylation of the gene promoter region was assessed using methylation-sensitive high-resolution melting (MS-HRM) [42,43].

CpG islands (regions of the genome that contain a large number of CpG dinucleotide repeats) in the promoters of the studied genes were predicted by the EMBOSS Cpgplot bioinformatics tool (https://www.ebi.ac.uk/Tools/seqstats/emboss_cpgplot/, Settings: Window: 100, Shift: 1, Obs./Exp.: 0.6, GC content: 50%). The primers were designed according to the instructions of Wojdacz et al. (2009) in MethPrimer (http://www.urogene.org/methprimer2/) [44]. Such a procedure necessitates DNA samples to be subjected to sodium bisulfite modification using a CiTi Converter DNA Methylation Kit (A&A Biotechnology, Gdynia, Poland), following the commercial instructions. A Bio-Rad CFX96 Real-Time PCR Detection System (BioRad Laboratories Inc., Hercules, CA, USA) was used for methylation analyses. Each PCR reaction consisted of 5x HOT FIREPol^®^ EvaGreen^®^ HRM Mix (no ROX) (Solis BioDyne, Tartu, Estonia), 500 nM of forward and reverse primers, and DNA samples (10 ng/µL) after bisulfite modification. The reaction conditions included initial activation, annealing, and elongation according to manufacturer’s instruction. However, the temperature of annealing was determined experimentally (see Table S2). The HRM analysis involved a step of denaturation at 95 °C for 15 s, reannealing at 60 °C for 1 min, and melting from 60 to 95 °C at a ramp rate of 0.2 °C every 2 s. The analysis of the obtained data was made using Bio-Rad Precision Melt Analysis Software (BioRad Laboratories Inc., Hercules, CA, USA). Thus, the methylation status was assessed by comparison of the HRM profiles obtained from the amplification of methylated DNA (CpGenome™ Rat Methylated Genomic DNA Standard, Merck Millipore Burlington, MA, USA) and unmethylated DNA (CpGenome™ Rat Unmethylated Genomic DNA Standard, Merck Millipore Burlington, MA, USA). To this end, serial dilutions were prepared: 0%, 10%, 25%, 50%, 75%, and 100% methylated DNA.

### 2.6. Western Blot Analysis

The protein levels of all the studied genes in the rat brain tissue were estimated using Western blotting analyses (Laemmli, 1970). Frozen brain structures samples were homogenized using a FastGene^®^ Tissue Grinder (Nippon Genetics Europe, Düren, Germany) in RIPA buffer (Radioimmunoprecipitation assay buffer) with 1 mM of phenylmethylsulfonyl fluoride as serine protease inhibitor. Then, the samples were sonicated twice and centrifuged, and supernatant was terminally collected. The protein concentration of received samples was estimated by the Lowry procedure [45]. Homogenate samples were run under reducing conditions on 10% SDS polyacrylamide gels and transferred onto Immobilon-P (Millipore, Bedford, MA, USA). Then, the blots were blocked with 5% non-fat dry milk in 0.1% TBST buffer (Tris-buffered saline with Tween-20) and after the block, the membranes incubated with the primary antibodies and the opportune secondary antibodies were conjugated with horseradish peroxidase (see Table S3). Finally, the blots were incubated with peroxidase substrate solution (Thermo Fisher Scientific, Waltham, MA, USA), and the proteins were visualized on X-ray film by enhanced chemiluminescence. Densitometry analysis of protein bands was using by Gel-Pro^®^ Analyzer Software (Media Cybernetics Inc., Silver Spring, MD, USA). The integrated optical density (IOD) of the immunoreactivity bands was measured based on digital images. Results were normalized using the reference protein (β-actin) resolved by electrophoresis.

### 2.7. Statistical Analysis

The results were presented as the mean value ± standard error of the mean. Data of the effect of 2 weeks of CMS on sucrose consumption were analyzed by using *test t*. The data of gene expression and methylation were analyzed using a two-way analysis of variance (two-way ANOVA) with *post hoc* Tukey’s test *p* values < 0.05 were considered as statistically significant. All the analyses were completed using Statistica 12 (Statsoft, Tulsa, OK, USA), SigmaPlot 11.0 (Systat Software Inc., San Jose, CA, USA) and GraphPad Prism 5.0 (GraphPad Software, Inc., La Jolla, CA, USA).

## 3. Results

### 3.1. Correlation between Differences in Sucrose Intake of Rats Subjected to Stress and Agomelatine Treatment

As shown in Table S4, before the stress was initiated (Week 0), the consumption of 1% sucrose solution was comparable in all groups. The intake decreased by approximately 50% (*p* < 0.01) after two weeks of the CMS procedure. A similar effect was observed in Stressed/Saline and Stressed/Ago groups (*p* < 0.05, *p* < 0.01, respectively). Chronic (five-week) administration of agomelatine had no effect in the case of control animals but normalized the intake in the stressed rats (*p* < 0.001). 

### 3.2. mRNA Expression Level of the Studied Genes

#### 3.2.1. Gene Expression in PBMCs and Brain Structures

**Figure 1:**
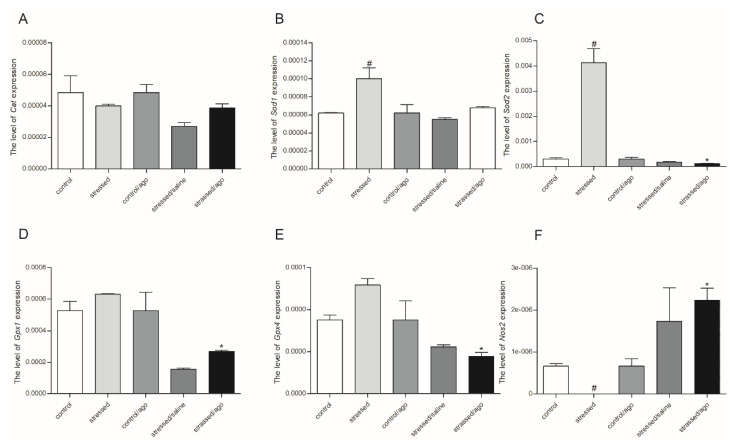
CAT - catalase (**A**) SOD1 – superoxide dismutase 1 (**B**), SOD2 – superoxide dismutase 2 (**C**), Gpx1 – glutathione peroxidase 1 (**D**), Gpx4 - glutathione peroxidase 4 (**E**), and NOS2 nitric oxide synthetase 2 (**F**) mRNA expression in peripheral blood mononuclear cells (PBMCs) of rats exposed to chronic mild stress (CMS) procedure for two weeks (Control, Stressed) and in animals exposed to CMS for seven weeks and administered vehicle (1 mL/kg) or agomelatine (10 mg/kg) for five weeks (Control/Ago, Stressed/Saline, Stressed/Ago). Relative mRNA expression was calculated using a 2^−ΔCt (Ctgene–Ct18S)^ method. Data were presented as means ± SEM. N = 6. * p <0.05 for differences between stressed and stressed/ago groups, and ^#^ p < 0.05 for differences between controls and stressed groups.

**Figure 2:**
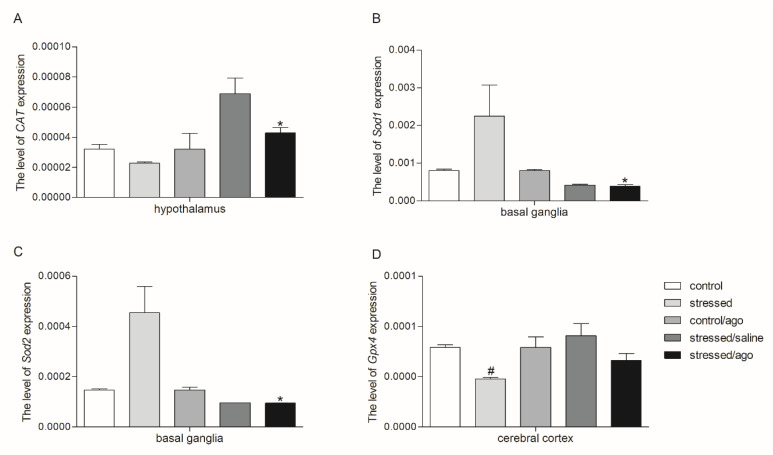
*CAT* (**A**) *SOD1* (**B**), *SOD2* (**C**), *Gpx1* (**D**), *Gpx4* (**E**), and *NOS1* (**F**) mRNA expression in the hippocampus, amygdala, hypothalamus, midbrain, cerebral cortex and basal ganglia of animals exposed to CMS procedure for two weeks (Control, Stressed) and in animals exposed to CMS procedure for seven weeks and administered vehicle (1 mL/kg) or agomelatine (10 mg/kg) for five weeks (Control/Ago, Stressed/Saline, Stressed/Ago). Relative mRNA expression was estimated using a 2^−ΔCt (Ctgene–Ct18S)^ method. Data were presented as means ± SEM. N = 6. * *p* < 0.05 for differences between stressed and stressed/ago groups, and ^#^ *p* < 0.05 for differences between controls and stressed groups.

As shown in Figure 1, Figure 2, and Figure S1, the effect of CMS and agomelatine on *Sod1*, *Sod2*, *Gpx1*, *Gpx4*, *Cat*, and *Nos1* mRNA expression depended on the tissue and brain structure. Agomelatine reduced the expression of *Sod1* and *Sod2* in the basal ganglia (*p* < 0.05). Similarly, in the case of PBMCs, reduced expression of *Sod2, Gpx1*, *Gpx4* (*p* < 0.05) was observed after agomelatine. Additionally, the CMS procedure increased the expression of *Sod2* in PBMCs (*p* < 0.05). On the other hand, the expression of *CAT* in the hypothalamus (*p* < 0.05) and *Nos2* in PBMCs (*p* < 0.05) was significantly increased as an effect of the antidepressant therapy in the stressed rats. Moreover, the cerebral cortex of the stressed group was characterized by reduced expression of *Gpx4* (*p* < 0.05), whereas *Sod1* expression in PBMCs was elevated in the stressed rats (*p* < 0.05), but *Nos2* expression was decreased in the PBMCs of stressed rats.

#### 3.2.2. Comparison of Agomelatine Impact on Gene Expressions in PMBCs and Brain Structure

As shown in Supplementary Figure S2, agomelatine caused a drop of *Gpx4* expression in the hypothalamus and cerebral cortex and increased the gene expression in PBMCs (*p* < 0.001). No significant differences were detected in the case of other studied genes.

### 3.3. Methylation Changes in the Studied Gene Promotors

#### 3.3.1. Methylation Status of Gene Promoters in Peripheral Blood Mononuclear Cells and Brain Structures

**Figure 3:**
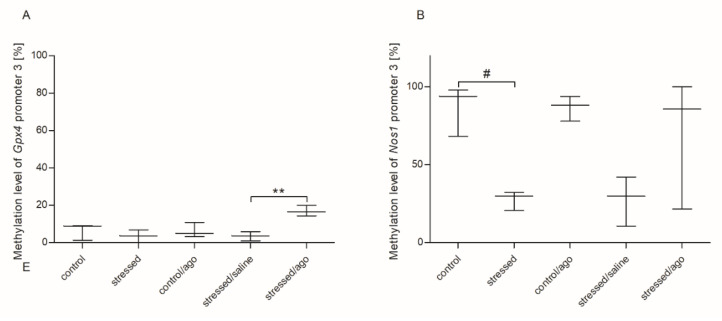
The methylation status *Gpx4* promoter 3 (**A**), *NOS1* promoter 3 (**B**), in PBMCs of animals exposed to CMS procedure for two weeks (Control, Stressed) and in animals exposed to CMS procedure for seven weeks and administered vehicle (1 mL/kg) or agomelatine (10 mg/kg) for five weeks (Control/Ago, Stressed/Saline, Stressed/Ago). Data presented as means ± SEM. N = 6. ** *p* < 0.01 for differences between stressed and stressed/ago groups, and ^#^ *p* < 0.05 for differences between controls and stressed groups.

**Figure 4:**
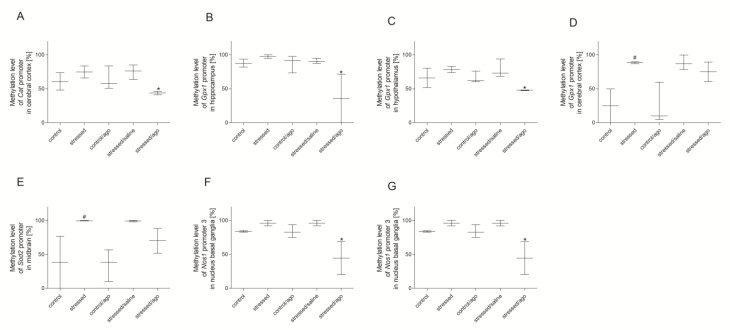
The methylation status of *CAT* promoter in cerebral cortex (**A**), *Gpx1* promoter in hippocampus (**B**), hypothalamus (**C**), and cerebral cortex (**D**), *SOD2* promoter in midbrain (**E**), *NOS1* promoter 3 in (**F**) and amygdala (**G**) of animals exposed to CMS procedure for two weeks (Control, Stressed) and in animals exposed to CMS procedure for seven weeks and administered vehicle (1 mL/kg) or agomelatine (10 mg/kg) for five weeks (Control/Ago, Stressed/Saline, Stressed/Ago). Data presented as means ± SEM. N = 6. * *p* < 0.05 for differences between stressed and stressed/ago groups, and ^#^ *p* < 0.05 for differences between controls and stressed groups.

As shown as Figure 3, the methylation status of the third *Nos1* promoter in PBMCs was significantly decreased in the stress group (*p* < 0.05) and the methylation of the third *Gpx4* promoter was increased in the stressed animals after agomelatine administration (*p* < 0.05). As shown in Figure 4, the CMS procedure significantly increased the methylation level of promoter 2 of *Gpx1* in the cerebral cortex (*p* < 0.05). Agomelatine significantly reduced the methylation status of *Nos1* promoter 3 in the amygdala and basal ganglia (test *p* < 0.05), *Gpx1* promoter in the hippocampus (*p* < 0.05) and hypothalamus (*p* < 0.05), as well as *Cat* promoter in the cerebral cortex (*p* < 0.05). Moreover, the methylation status of the *Sod2* promoter region in the midbrain was elevated in the stressed rats compared to the controls (*p* < 0.05). No significant differences were detected in the case of other promoter regions of the studied genes (Figure S3 and Table S4).

#### 3.3.2. Comparison of Agomelatine Impact on the Methylation Status of Gene Promoters in PBMCs and Brain Structure

As shown in Figure S4, significant differences were found in the effect of agomelatine on the methylation status of *Gpx4* promoter 3, *Sod2* promoter, and *Nos1* promoter 3 between blood and brain structures. In particular, agomelatine increased the methylation level of *Sod2* promoter in the hypothalamus and cerebral cortex compared to blood (*p* < 0.05, *p* < 0.01, respectively). Furthermore, an antidepressant therapy decreased the methylation of the *Nos1* promoter 3 region in the midbrain and basal ganglia compared to blood (*p* < 0.05). Additionally, the hippocampus, hypothalamus, midbrain, and cerebral cortex showed lower methylation levels of the *Gpx4* promoter 3 region than blood (*p* < 0.001).

### 3.4. Gene Expression at Protein Level

**Figure 5:**
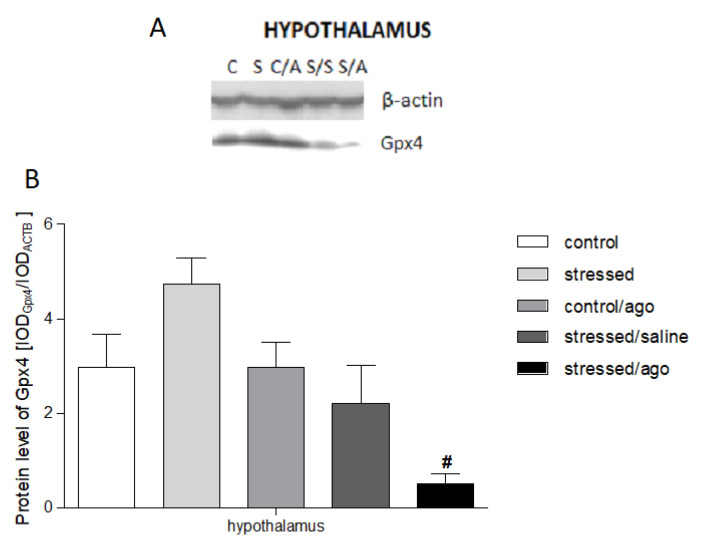
Gpx4 protein expression in the brain structures—hippocampus, amygdala, hypothalamus, midbrain, cerebral cortex and basal ganglia—of animals exposed to CMS procedure for two weeks (Control, Stressed) and in animals exposed to CMS procedure for seven weeks and administered vehicle (1 mL/kg) or agomelatine (10 mg/kg) for five weeks (Control/Ago, Stressed/Saline, Stressed/Ago). (**A**) Representative Western blot analysis in midbrain and cerebral cortex. C—controls, S—stressed for two weeks, C/A—Control/Agomelatine, S/S—Stressed/Saline, S/A—Stressed/Agomelatine. (**B**) Gpx4 protein level measured in all studied brain structures. The graphs show the mean integrated optical density (IODs) of the bands from all analyzed samples. The relative protein expression levels was calculated using the IOD_gene_/IOD_ACTB_ method. Data are presented as means ± SEM. N = 6. # *p* < 0.05 for the difference between stressed and stressed/ago groups.

The protein expression level of Sod1, Nos1, Nos2, Gpx4, and Cat did not differ between the studied groups (Figure S5). As shown in Figure 5, agomelatine significantly reduced only the level of Gpx4 protein in the hypothalamus (*p* < 0.05).

## 4. Discussion

Our study presents effects of antidepressant treatment with agomelatine on the disorders of the selected genes encoded antioxidant enzymes in the PBMCs and various brain structures (hippocampus, amygdala, hypothalamus, midbrain, cerebral cortex, basal ganglia) of the rats exposed to the chronic mild stress procedure. As in previous studies, the CMS procedure caused a significant (approximately 50%) reduction of the consumption of 1% sucrose solution, indicating a generalized deficit in sensitivity to reward in the stressed animals, i.e., a characteristic symptom of depression [46,47]. This effect was normalized by a chronic (5 weeks) administration of agomelatine, which did not affect the behavior of control, non-stressed animals. The CMS procedure and agomelatine treatment also influenced mRNA and protein levels and the status of promoter region methylation of selected genes encoding enzymes involved in oxidative stress in the PBMCs and in various brain structures. Overall, the gene changes seem to confirm earlier suggestions that the overproduction of reactive oxygen species leads to development of depression-like symptoms in the CMS rats [4,5,48] as well as to increases in the level of thiobarbituric acid-reactive substances (TBARS), lipid peroxidation, protein oxidation, and oxidative DNA damage in several brain structures [49,50,51]. These findings are consistent with clinical reports showing that elevated plasma levels of reactive oxygen species (ROS) and decreased activity of antioxidant enzymes are observed in patients suffering from depression [52].

Demirdaş et al. (2016) showed that the activity of Gpx and glutathione level in the brain and other organs were lower in the CMS animals compared to non-stressed rats, but they were higher in the agomelatine-treated group [53]. Moreover, an in vitro study conducted on a neuronal cell line confirmed that agomelatine caused an increase in glutathione level and glutathione peroxidase activity [54]. Thus, the CMS animal model depression may be associated with the intensification of oxidative stress and reduced antioxidant levels, both effects being normalized by agomelatine. On the other hand, in our study, agomelatine decreased levels of *Gpx1* and *Gpx4* expression in PBMCs and reduced the CMS-induced enhancement of *Gpx4* expression at the protein level in the hippocampus. The decreased level of *Gpx1* and *Gpx4* expression in stressed animals after antidepressant therapy may be a result of reduced reactive oxygen species level caused by chronic agomelatine administration. The reduced ROS concentration after therapy may cause a decreased demand for antioxidative defense enzymes [55]. Other results showed that melatonin receptor agonists are characterized by ROS scavenging activity and antioxidant system modulation [56] and that the elevated expression of antioxidant enzymes is associated with the activation of melatonin receptors [34]. Differences between the analyzed studies may result from the dualistic model of the agomelatine action. Agomelatine may act as a free radical scavenger by acting directly on the ROS level or it may indirectly affect the activity of antioxidant enzymes. In the first case, the enzyme activity decreases as a result of lowering the production level of ROS. In turn, in the second case, increasing the activity of antioxidant enzymes caused by agomelatine leads to a decrease of ROS level. Similarly, in the case of human study, the obtained results are divergent. On the one hand, the depression may be associated with the decreased activity of GPx and SOD [15,16,17]. On the other hand, Gałecki et al. and Kodydková et al. reported the opposite effect [7,8].

The next antioxidant enzyme studied in this study was catalase, which is an enzyme that was shown to be substantially increased in the serum of depressed patients [57]. In addition, in CMS animals, the activity of Cat was elevated in various brain structures [51]. The increased Cat activity in stressed animals and depressed patients is a consequence of increased hydrogen peroxide production. The previous studies showed that depression is associated with an increased activity of xanthine oxidase, which generated hydrogen peroxide. Then, hydrogen peroxide may be neutralized by catalase into water and oxygen [16,18,19]. The recent study showed that agomelatine administration caused an increase in catalase activity in the prefrontal cortex, hippocampus, and striatum of mice after chemically induced seizures [55]. In this study, we also found an increased expression of *Cat* in the hypothalamus and reduced methylation status of the *Cat* promoter region in the cerebral cortex in the agomelatine-treated CMS rats. Mello et al. (2016) demonstrated that agomelatine increased Sod activity in the striatum, whereas Cat activity was reduced in the cerebellum and elevated in the posterior cortex [58]. Thus, the results suggest that agomelatine may modulate the expression and promoter methylation of gene-encoding Cat. The chronic administration of agomelatine may cause an increase of Cat expression and decrease methylation in the Cat promoter region, consequently contributing to the increase in the activity of this enzyme and reducing the concentration of hydrogen peroxide. An interesting relationship has been observed in the case of Sod expression. Sod catalyzes the dismutation of the superoxide radical into ordinary molecular oxygen and hydrogen peroxide [56]. In the PBMCs of stressed rats, the Sod1 and Sod2 mRNA expression were increased, whereas the chronic administration of agomelatine normalized this effect. Moreover, agomelatine treatment reduced Sod1 and Sod2 expression in the basal ganglia of the stressed rats. Similarly, in depressed patients, the serum activity of SOD was higher than in control subjects [56]. In this connection, that superoxide radical excess is neutralized by Sod, while the increased expression level and activity of Sod were observed in stressed rats and depressed patient. However in animal models, the activity of this enzyme was decreased both in serum and in brain structures [51,59].

The next enzyme associated with mechanisms of depression and nitrosative stress is Nos. Depressed patients are characterized by elevated serum levels of NO and its metabolites [60,61,62], and these effects were normalized by antidepressant therapy [60]. In addition, in mice exposed to the CMS model, the production of NO was increased [59]. In this study, we found that the CMS caused reduction of the methylation status of the third *NOS1* promoter region in PBMCs, whereas the methylation level of the third and seventh *Nos1* promoters in the basal ganglia and the expression of *Nos2* in PBMCs after agomelatine therapy were lower than in the saline-treated stressed animals. There are reports suggesting that melatonin plays an important role in NO generation; the compound was found to inhibit constitutive NOS (NOS1) and to control the mitochondrial isoform of NOS [63,64]. Consequently, it can be assumed that agomelatine exhibits antioxidant activity by modulating nitric oxide levels by the stimulation of melatonin MT1 receptors [60,65].

These findings seem to confirm the idea that insufficient antioxidant defense may be associated with depression-like symptoms observed in animals after the CMS procedure. Moreover, it may be suggested that the antioxidant imbalance causes an overproduction of toxic free radicals, leading to serious damage to biomolecules, including lipids, proteins, and DNA, contributing to various diseases. Long-term exposure to free radicals may consequently cause cell death as well as neuronal and glia atrophy, leading to a decreased volume of gray matter and reduced number of cells observed in depressed patients [66,67]. All these effects are attenuated by antidepressant drugs, which may recover the oxidative and antioxidative balance by normalizing the expression level of antioxidant enzymes.

Together, these studies indicate that agomelatine may prevent the ROS overproduction in animals exposed to the CMS procedure. The results also suggest that agomelatine has antioxidant activity, and this may be involved in the mechanisms of its relief of depression-like symptoms. Agomelatine reduces the mRNA expression of Sod2, Gpx1, Gpx4, and Nod2 in PBMCs, and Sod1 and Sod2 in the basal ganglia. The reduced expression level of the antioxidant enzyme may be associated with a decreased generation of free radicals after agomelatine therapy, and therefore, the expression level of genes involved in oxidative stress could be an indicator of its antidepressant effectiveness. On the other hand, the presented study has some limitations. First, only the effects of CMS and agomelatine treatment were analyzed without assessing the effects of other biochemical pathways, including inflammation or the tryptophan catabolites (TRYCATs) pathway. Previous studies confirmed that depression is a multifactorial disease. Both pro-inflammatory factors and toxic metabolites of the TRYCATs pathway can additionally increase the production of reactive oxygen species. Therefore, in the future, other biochemical pathways that may have an impact on the development of oxidative stress should also be analyzed. On the other hand, elements of the pathways that are affected by the overproduction of reactive oxygen species, including pathways for repairing oxidative DNA damage, should also be investigated [20,68,69]. Another limitation is the limited possibility to extrapolate animal broadcast results to changes observed in humans. Therefore, the next step of the study should be the analysis of changes in the studied genes in patients with depression.

## 5. Conclusions

The presented results confirm the hypothesis that the oxidative and nitrosative stress is involved in the pro-depressive effects of the CMS procedure and in the mechanisms of therapeutic action of agomelatine in this model of depression: (1) CMS causes the modulation of *Sod1* expression in PBMCs and Gpx4 in the brain; (2) the administration of agomelatine modifies the expression of *Sod2*, *Gpx1*, *Gpx4,* and *Nos2* in PBMCs, and *Sod1* and *Sod2* in the brain; (3) CMS and agomelatine change the methylation status of *Gpx1, Gpx4,* and *Nos1* promoter in PBMCs and in the brain. Interestingly, the presented results suggested that agomelatine may have various mechanisms of action. The treatment with agomelatine may directly impact of ROS concentration or may indirectly modulate the gene expression, methylation, and activity of antioxidant enzymes. Thus, further research is needed to explain the cause of this phenomenon. It is possible that other biochemical pathways that affect the intensification of oxidative stress have a decisive impact on the antidepressant action of agomelatine.

## Data Availability

The data that support the findings of this study are available from the corresponding author upon reasonable request.

## Supplementary Materials

The following are available online at https://www.mdpi.com/2073-4425/11/6/644/s1. Table S1: Characteristics of the genes studied (All data contained in the table were compiled with the help of Genomatix Software Suite, Intrexon Bioinformatics Germany GmbH, Munich, Germany, 2019), Table S2: Conditions of the antibodies used in the Western blot analysis, Table S3: The characteristics of primers used for analysis of methylation levels in the promoter regions of the studied genes, Table S4: The effects of chronic mild stress and treatment on sucrose intake in controls, depressed rats and rats after treatment with venlafaxine (N = 6), Table S5: The methylation status of CAT promoter (A) Gpx1 promoter (B), Gpx4 promoter 2 (C) Gpx4 promoter 3 (D) SOD1 (E), SOD2 (F) NOS1 promoter 3 (G), NOS1 promoter 7 (H) in hippocampus, amygdala, hypothalamus, midbrain, cortex and basal ganglia of animals exposed to CMS procedure for two weeks (Control, Stressed) and in animals exposed to CMS procedure for seven weeks and administered vehicle (1 ml/kg) or agomelatine (10 mg/kg) for five weeks (Control/Ago, Stressed/Saline, Stressed/Ago), Figure S1: Differences in CAT (A) SOD1 (B), SOD2 (C), Gpx1 (D), Gpx4 (E) gene expression between hippocampus, amygdala, hypothalamus, midbrain, cortex and basal ganglia and PBMCs of animals exposed to CMS procedure for two weeks (Control, Stressed) and in animals exposed to CMS procedure for seven weeks and administered vehicle (1 ml/kg) or agomelatine (10 mg/kg) for five weeks (Control/Ago, Stressed/Saline, Stressed/Ago), Figure S2: CAT (A), SOD1 (B), SOD2 (C), Gpx1 (D), Gpx4 (E) mRNA expression in PBMCs and in the hippocampus, amygdala, hypothalamus, midbrain, cortex and basal ganglia of animals exposed to CMS procedure for two weeks (Control, Stressed) and in animals exposed to CMS procedure for seven weeks and administered vehicle (1 ml/kg) or agomelatine (10 mg/kg) for five weeks (Control/Ago, Stressed/Saline, Stressed/Ago), Figure S3: The methylation status of Cat promoter (A) Sod1 (B), Gpx1 (C), NOS1 promoter 7 (D) in PBMCS of animals exposed to CMS procedure for two weeks (Control, Stressed) and in animals exposed to CMS procedure for seven weeks and administered vehicle (1 ml/kg) or agomelatine (10 mg/kg) for five weeks (Control/Ago, Stressed/Saline, Stressed/Ago), Figure S4: Differences in the methylation status of CAT (A), Gpx1 (B) Gpx4 promoter 2 (C) promoter 3 (D), SOD1 (E), SOD2 (F) and NOS1 promoter 3 (G), promoter 7 (H) between hippocampus, amygdala, hypothalamus, midbrain, cortex and basal ganglia and PBMCs of animals exposed to CMS procedure for two weeks (Control, Stressed) and in animals exposed to CMS procedure for seven weeks and administered vehicle (1 ml/kg) or agomelatine (10 mg/kg) for five weeks (Control/Ago, Stressed/Saline, Stressed/Ago), Figure S5: SOD1 (A) and NOS1 (B) protein expression in animals exposed to CMS procedure for two weeks (Control, Stressed) and in animals exposed to CMS for seven weeks and administered vehicle (1 ml/kg) or agomelatine (10 mg/kg) for five weeks (Control/Ago, Stressed/Saline, Stressed/Ago).
