# Supplementary material for: The Changes of Expression and Methylation of Genes Involved in Oxidative Stress in Course of Chronic Mild Stress and Antidepressant Therapy with Agomelatine
Source: Genes (Basel). 2020 Jun 11;11(6):644. doi: 10.3390/genes11060644 (PMC7349414; doi:10.3390/genes11060644)
Supplement: Supplementary file 1 [file genes-11-00644-s001.pdf]

genes

MDPI

Supplementary Materials: The Changes of Expression and Methylation of Genes Involved in Oxidative Stress in Course of Chronic Mild Stress and Antidepressant Therapy with Agomelatine

Table S1. Characteristics of the genes studied (All data contained in the table were compiled with the help of Genomatix Software Suite, Intrexon Bioinformatics Germany GmbH, Munich, Germany, 2019).

Gene name	Function of protein encoding the gene	Chromosomal location	Biased expression
Catalase (*Cat*)	hydrogen peroxide reductase	3q32	Adrenal, kidney, spleen, thymus, heart, liver
Glutathione peroxidase 1 (*Gpx1*)	Catalyses reduction of organic hydroperoxides and hydrogen peroxide by glutathione and thereby protect cells from oxidative damage	8q32	Adrenal, heart, kidney, liver, lung, muscle, spleen, thymus, uterus
Glutathione peroxidase 4 (*Gpx4*)	Catalyses reduction of hydrogen peroxide, organic hydroperoxides and lipid hydroperoxides, and thereby protect cells from oxidative damage	7q11	Adrenal, brain, heart, kidney, muscle, testes
superoxide dismutase 1 (*Sod1*)	Catalyzes conversion of supeoxide to hydrogen peroxide and molecular oxygen, involved in response to oxidative stress	11q11	Adrenal, brain, heart, kidney, liver, lung, muscle, spleen, testes, thymus, uterus
Superoxide dismutase 2 (*Sod2*)	Intramitochondrial free radical scavenging enzyme	1q11	Adrenal, brain, heart, kidney, liver, muscle
Nitric oxide synthase 1 (*Nos1*)	Catalyzes production of nitric oxide	12q16	Adrenal, brain, kidney, muscle
Nitric oxide synthase 2 (*Nos2*)	Cytokine-inducible enzyme involved in nitric oxide production	10q25	Adrenal, heart, lung, muscle, spleen, thymus

Table S2. Conditions of the antibodies used in the Western blot analysis.

	Primary antibody	Secondary antibody
**β-actin (a reference protein)**	mouse, 1:1000, (Santa Cruz Biotechnolgy Inc), 1 hour at room temperature	anti-mouse, 1:6000, (Cell Signalling Technologies Inc., Danvers, Massachusetts, USA), 1 hour at room temperature
**catalase**	mouse, 1:1000, (Santa Cruz Biotechnolgy Inc), overnight at 4°C	anti-mouse, 1:6000, (Cell Signalling Technologies Inc., Danvers, Massachusetts, USA), 1 hour at room temperature
**glutathione peroxidase 4**	rabbit, 1:6000, (Abcam), overnight at 4°C	anti-rabbit, 1:6000, (Cell Signalling Technologies Inc., Danvers, Massachusetts, USA), 1 hour at room temperature
**superoxide dismutase 1**	mouse, 1:1000, (Santa Cruz Biotechnolgy Inc), 2 hour at room temperature	anti-mouse, 1:6000, (Cell Signalling Technologies Inc., Danvers, Massachusetts, USA), 1 hour at room temperature

Table S3. The characteristics of primers used for analysis of methylation levels in the promoter regions of the studied genes.

Gene	Starter sequence	Product size	Tm
Cat	F:TTTGAGATTATTGTGTTTGAAA<br>R:TACCTACACCCAAAAAAAAATA	148	59
Gpx1	F:GTTGTTTTAGGTTTTGTTGTTG<br>R:AAAACTAAAATCCTCCAACTCT	102	65
Gpx4 (promotor 2)	F:AGGTTGGAGGTTTAGAGGTTTA<br>R:TCCCCTAAATACAAAAATCTCT	118	59
Gpx4 (promotor 3)	F:AGGTTGGAGGTTTAGAGGTTTA<br>R:AAAACATAACAAAATCATCTCCC	147	65
Sod1	F: AAGGAGGTGTGTTTAATTGGTA<br>R: AACCCCTCTCACAAATTTCTAA	144	65
Sod2	F: GGGGAAGGTTATTTAGGGTATA<br>R: CCTTTTCCATTCCTAATTCTAAA	133	59
Nos1 (promotor 3)	F: GGGTTTTTAATTTTTTTATTGTG<br>R: CAACCCTCATTAAAAAAACC	124	59
Nos1 (promotor 7)	F: GTTTGAGATTGGAATTTTTTGG<br>R: CCAAAACATCCAAAAATACACA	124	59

Table S4. The effects of chronic mild stress and treatment on sucrose intake in controls, depressed rats and rats after treatment with venlafaxine (N = 6). The data presented as mean ± SEM, ** $p < 0.01$ for the difference between control rats and stressed rats, *** $p < 0.001$ for the difference between rats before and after treatment in Stressed/Ago group, ## $p < 0.01$ for the difference between the rats before CMS procedure and groups after two-week stress in Stressed group, & $p < 0.05$ for the difference before and after the CMS procedure in the Stressed/Saline group.

Sucrose intake	Control	Stressed	Control/Ago	Stressed/Saline	Stressed/Ago
**Before CMS procedure**	9.85 ± 0.74	11.59 ± 1.04	12.90 ± 1.83	11.30 ±0.89	11.76 ± 0.64
**After two weeks of stress**	11.63 ± 1.13	5.73 ± 0.98 ##	13.27 ± 1.43	6.04 ± 1.44 &	6.50 ± 0.72 **
**After five weeks of agomelatine therapy**			12.46 ± 2.10	5.75 ± 0.44	12.32 ± 1.13 ***

Table S5. The methylation status of *CAT* promoter (A) *Gpx1* promoter (B), *Gpx4* promoter 2 (C) *Gpx4* promoter 3 (D) *SOD1* (E), *SOD2* (F) *NOS1* promoter 3 (G), *NOS1* promoter 7 (H) in hippocampus, amygdala, hypothalamus, midbrain, cortex and basal ganglia of animals exposed to CMS procedure for two weeks (Control, Stressed) and in animals exposed to CMS procedure for seven weeks and administered vehicle (1 ml/kg) or agomelatine (10 mg/kg) for five weeks (Control/Ago, Stressed/Saline, Stressed/Ago). Data presented as means ± SEM. N = 6. No significant changes were found between any groups.

(A) **Methylation level of *CAT* promoter**

Part of brain	Control	Stressed	Control/Ago	Stressed/Saline	Stressed/Ago
Hippocampus	87.62 ± 2.11	82.93 ± 5.66	77.25 ± 11.11	71.31 ± 11.62	20.08 ± 8.68
Amygdala	98.34 ± 0.93	88.77 ± 6.48	79.43 ± 10.31	78.33 ± 13.81	51.02 ± 9.65
Hypothalamus	99.41 ± 0.31	79.27 ± 11.97	99.33 ± 0.88	71.89 ± 9.89	41.32 ± 9.38
Cerebral cortex	60.78 ± 7.40	74.84 ± 5.08	59.53 ± 11.21	64.41 ± 12.80	43.71 ± 1.36
Basal ganglia	91.75 ± 4.76	98.04 ± 1.13	96.77 ± 3.21	99.01 ± 0.11	46.70 ± 6.83

(B) **Methylation level of *Gpx1* promoter**

Part of brain	Control	Stressed	Control/Ago	Stressed/Saline	Stressed/Ago
Amygdala	95.80 ± 2.26	87.73 ± 7.09	98.33 ± 1.11	91.54 ± 11.04	74.12 ± 14.94
Midbrain	33.89 ± 19.57	73.88 ± 1.53	27.84 ± 15.65	69.95 ± 11.87	50.00 ± 28.87
Basal ganglia	55.46 ± 25.71	98.45 ± 5.88	61.94 ± 13.89	98.33 ± 0.88	72.07 ± 16.13

(C) **Methylation level of *Gpx4* promoter 2**					
**Part of brain**	**Control**	**Stressed**	**Control/Ago**	**Stressed/Saline**	Stressed/Ago
Hippocampus	50.00 ± 28.87	96.67 ±1.92	45.98 ± 11.73	91.64 ±6.74	50.00 ± 28.87
Amygdala	98.43 ± 0.47	72.08 ± 16.12	98.33 ± 0.88	69.52 ± 9.63	50.00 ± 28.87
Hypothalamus	85.43 ± 8.41	99.21 ± 0.54	73.54 ± 14.48	99.33 ± 0.88	85.47 ± 8.39
Midbrain	50.00 ± 28.87	50.00 ± 28.87	49.33 ± 5.85	39.52 ± 19.78	58.03 ± 19.81
Cerebral cortex	0.00 ± 0.00	51.85 ± 6.91	10.00 ± 0.71	45.64 ± 19.71	83.91 ± 9.29
Basal ganglia	81.80 ± 10.51	98.76 ± 0.54	77.53 ± 7.52	98.33 ± 0.88	98.56 ± 0.76

**(D) Methylation level of *Gpx4* promoter 3**					
**Part of brain**	**Control**	**Stressed**	**Control/Ago**	**Stressed/Saline**	Stressed/Ago
Hippocampus	61.80 ± 1.06	72.33 ± 0.70	49.62 ± 21.11	62.33 ± 10.95	76.93 ± 0.85
Amygdala	74.76 ± 2.94	71.09 ± 4.95	69.63 ± 6.93	69.53 ± 7.43	92.15 ± 2.12
Hypothalamus	54.55 ±12.14	68.16 ± 2.37	59.62 ± 9.62	77.52 ± 12.95	70.33 ± 3.62
Midbrain	54.95 ± 8.45	75.28 ± 3.58	49.63 ± 11.63	69.89 ± 9.53	67.52 ± 4.52
Cerebral cortex	69.20 ± 0.42	66.45 ± 4.04	75.72 ± 7.52	59.62 ± 13.65	63.41 ± 2.59
Basal ganglia	67.10 ± 9.00	72.13 ± 4.04	57.11 ± 19.83	79.73 ± 9.04	88.90 ± 0.06

**(E) Methylation level of *SOD1* promoter**					
**Part of brain**	**Control**	**Stressed**	**Control/Ago**	**Stressed/Saline**	Stressed/Ago
Hippocampus	39.07 ± 0.27	37.00 ± 1.64	45.64 ± 9.65	32.74 ± 6.48	36.04 ± 3.61
Amygdala	36.44 ± 5.32	46.22 ± 4.13	42.85 ± 7.75	52.67 ± 9.33	19.50 ± 12.50
Hypothalamus	40.26 ± 2.49	47.20 ± 2.03	38.74 ± 7.93	49.29 ± 6.55	40.64 ± 3.33
Midbrain	39.67 ±3.29	39.76 ± 0.94	45.89 ± 8.54	45.68 ± 3.89	44.96 ± 1.90
Cerebral cortex	45.36 ± 0.14	49.60 ± 6.30	35.75 ± 9.74	53.33 ± 8.83	46.13 ± 5.01
Basal ganglia	33.01 ± 5.09	38.18 ± 4.92	39.76 ± 6.87	40.91 ± 6.21	49.96 ±8.52

**(F) Methylation level of *SOD2* promoter**					
**Part of brain**	**Control**	**Stressed**	**Control/Ago**	**Stressed/Saline**	Stressed/Ago
Hippocampus	59.61 ± 2.85	53.59 ± 13.91	55.74 ± 5.66	49.73 ± 6.52	50.86 ± 18.51
Amygdala	58.64 ± 0.08	93.69 ± 3.64	64.64 ± 9.59	95.61 ± 13.55	76.97 ± 13.30
Hypothalamus	50.00 ±28.87	47.98 ± 14.24	49.01 ± 7.52	53.62 ± 9.67	78.76 ± 12.26
Cerebral cortex	19.51 ± 9.35	14.84 ± 0.49	25.75 ± 15.87	19.45 ± 6.78	66.82 ± 5.64
Basal ganglia	53.32 ± 26.95	99.94 ± 0.04	59.49 ± 5.76	99.33 ± 0.88	69.14 ± 17.82

**(G) Methylation level of *NOS1* promoter 3**					
**Part of brain**	**Control**	**Stressed**	**Control/Ago**	**Stressed/Saline**	Stressed/Ago
Hippocampus	87.23 ± 7.37	89.56 ± 6.03	89.63 ± 5.32	92.84 ± 11.16	83.29 ± 4.00
Hypothalamus	90.57 ± 2.79	86.59 ± 0.35	89.83 ± 6.53	81.63 ± 9.74	67.54 ± 0.01
Midbrain	87.04 ± 7.48	81.83 ± 10.49	92.73 ± 13.83	81.83 ± 15.33	26.22 ± 3.97
Cerebral cortex	59.88 ± 0.69	70.92 ± 1.53	65.84 ± 7.93	70.92 ± 11.73	98.13 ± 6.34

**(H) Methylation level of *NOS1* promoter 7**					
**Part of brain**	**Control**	**Stressed**	**Control/Ago**	**Stressed/Saline**	Stressed/Ago
Hippocampus	98.33 ± 0.88	98.88 ± 0.55	95.52 ± 7.63	98.33 ± 0.88	90.20 ± 0.65
Amygdala	97.67 ± 1.20	79.96 ± 11.57	91.83 ± 9.83	82.73 ± 15.93	88.65 ± 6.55
Hypothalamus	98.33 ± 0.88	75.28 ± 14.27	95.93 ± 0.98	80.11 ± 21.19	98.33 ± 0.88
Midbrain	85.30 ± 2.67	97.69 ± 0.87	79.83 ± 7.98	91.93 ± 3.73	98.33 ± 0.88
Cerebral cortex	71.65 ± 16.37	57.90 ± 23.04	69.93 ± 11.83	62.94 ± 26.99	57.83 ± 24.35
Basal ganglia	99.50 ± 0.41	98.33 ± 0.53	99.33 ± 0.88	98.33 ± 0.88	99.04 ± 0.32

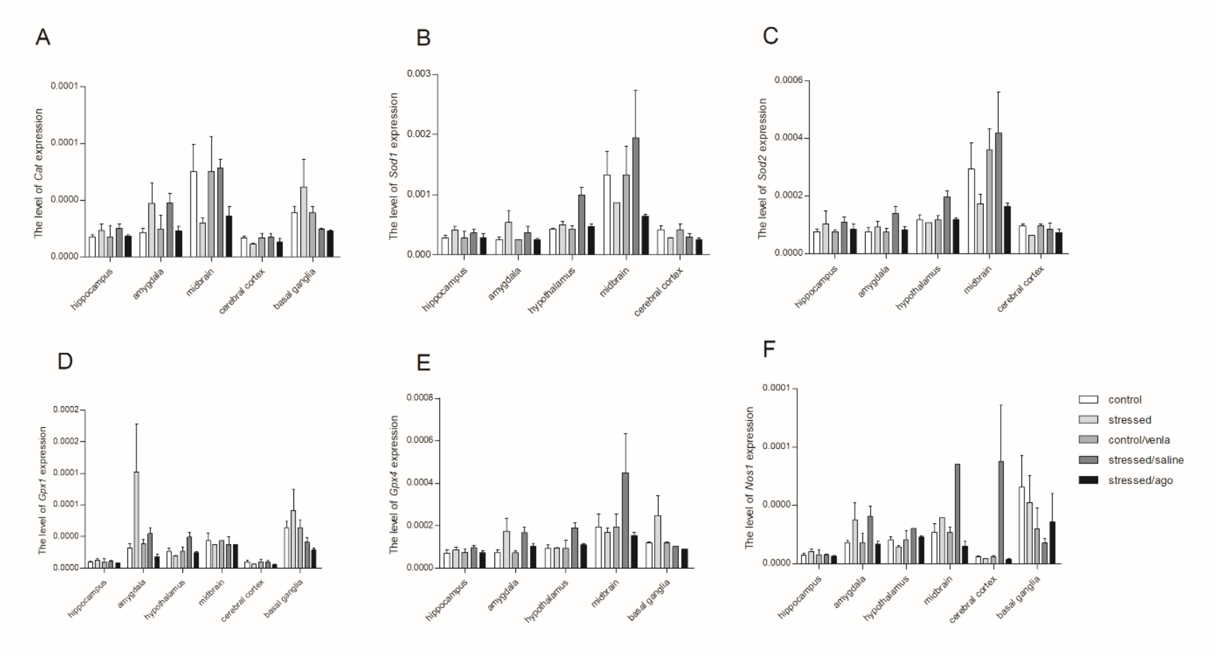

Figure S1. Differences in *CAT (A) SOD1 (B), SOD2 (C), Gpx1 (D), Gpx4 (E)* gene expression between hippocampus, amygdala, hypothalamus, midbrain, cortex and basal ganglia and PBMCs of animals exposed to CMS procedure for two weeks (Control, Stressed) and in animals exposed to CMS procedure for seven weeks and administered vehicle (1 ml/kg) or agomelatine (10 mg/kg) for five weeks (Control/Ago, Stressed/Saline, Stressed/Ago). Relative mRNA expression were estimated using a $2^{-\Delta Ct\ (Ct\ gene - Ct\ 18S)}$ method. Data presented as means ± SEM. N = 6. * $p < 0.05$, ** $p < 0.01$, *** $p < 0.001$.

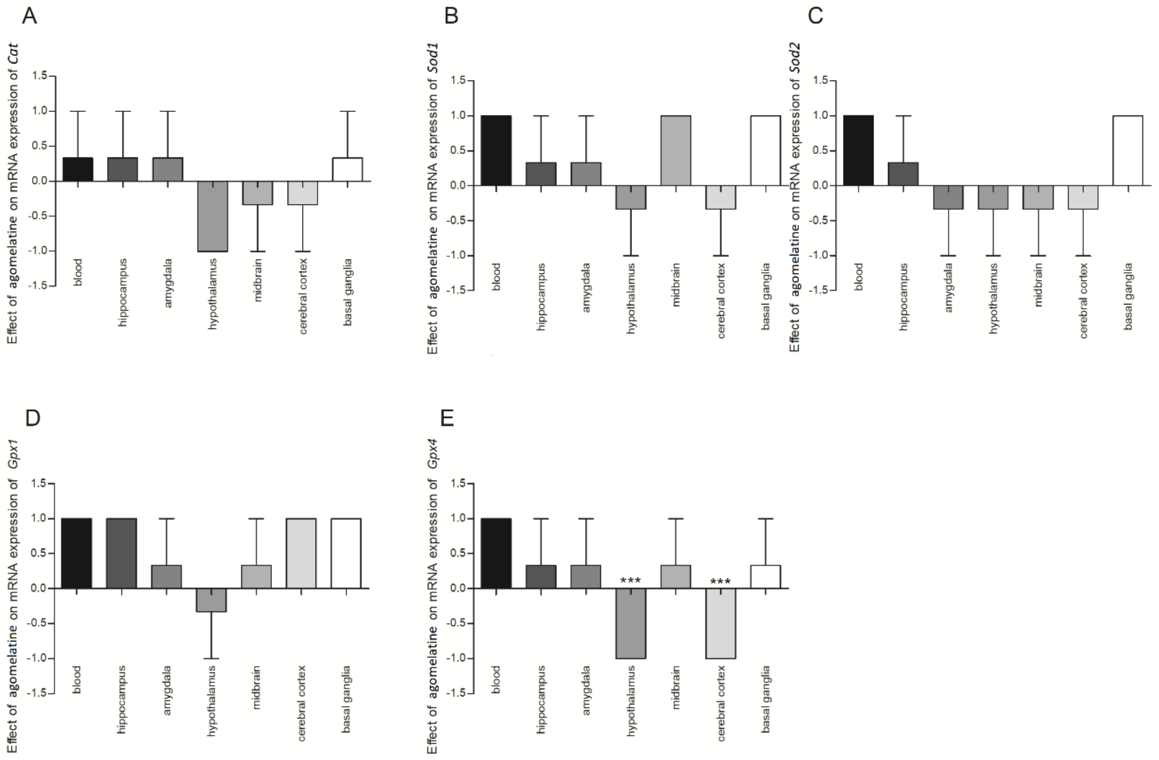

Figure S2. *CAT* (A), *SOD1* (B), *SOD2* (C), *Gpx1* (D), *Gpx4* (E) mRNA expression in PBMCs and in the hippocampus, amygdala, hypothalamus, midbrain, cortex and basal ganglia of animals exposed to CMS procedure for two weeks (Control, Stressed) and in animals exposed to CMS procedure for seven

weeks and administered vehicle (1 ml/kg) or agomelatine (10 mg/kg) for five weeks (Control/Ago, Stressed/Saline, Stressed/Ago). The effects are presented as fold change ($2^{-\Delta\Delta Ct}$ method; Schmittgen and Livak, 2008). Data presented as means ± SEM. N = 6. *** $p < 0.001$ for differences between blood and all studied brain structures.

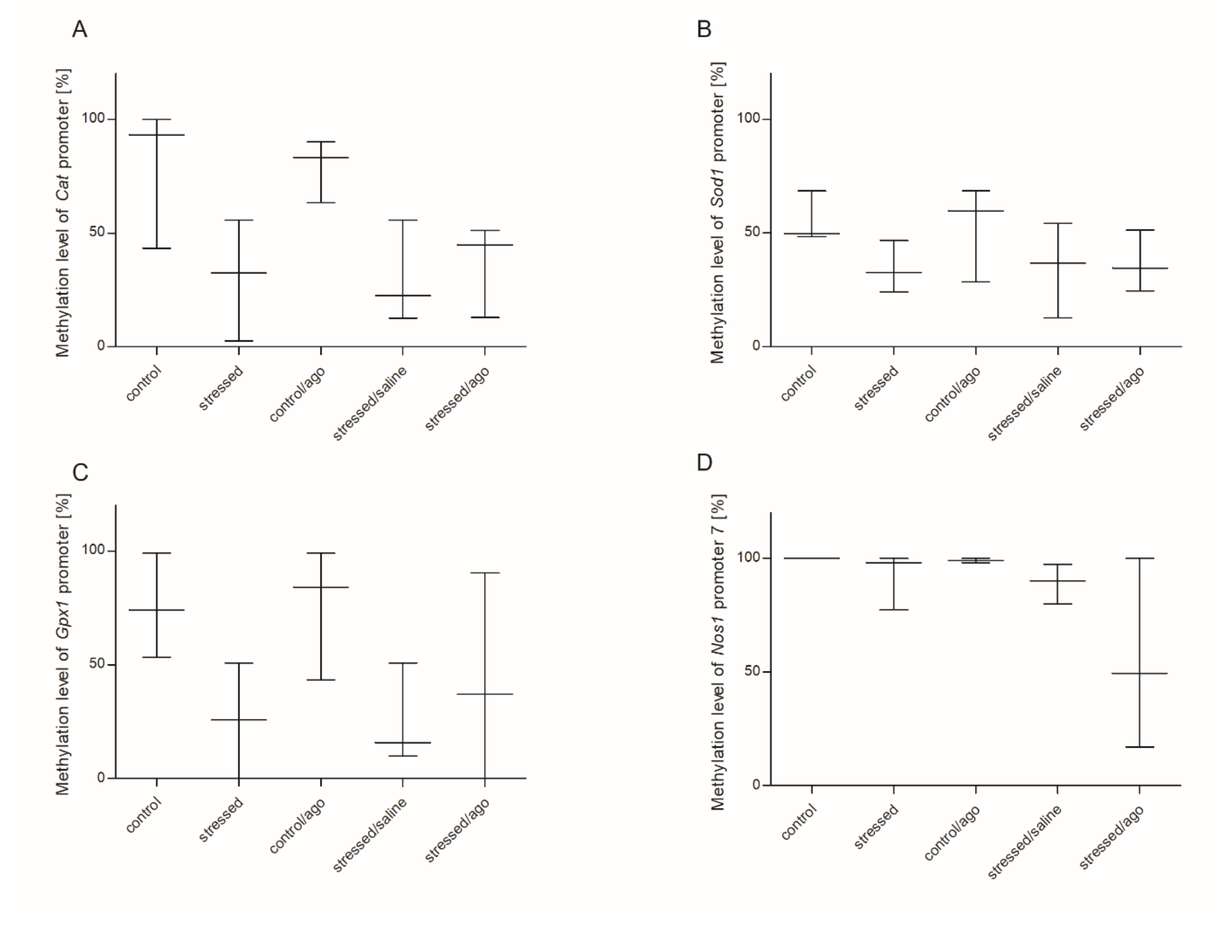

Figure S3. The methylation status of *Cat* promoter (A) *Sod1* (B), *Gpx1* (C), *NOS1* promoter 7 (D) in PBMCS of animals exposed to CMS procedure for two weeks (Control, Stressed) and in animals exposed to CMS procedure for seven weeks and administered vehicle (1 ml/kg) or agomelatine (10 mg/kg) for five weeks (Control/Ago, Stressed/Saline, Stressed/Ago). Data presented as means ± SEM. N = 6. No significant changes were found between any groups.

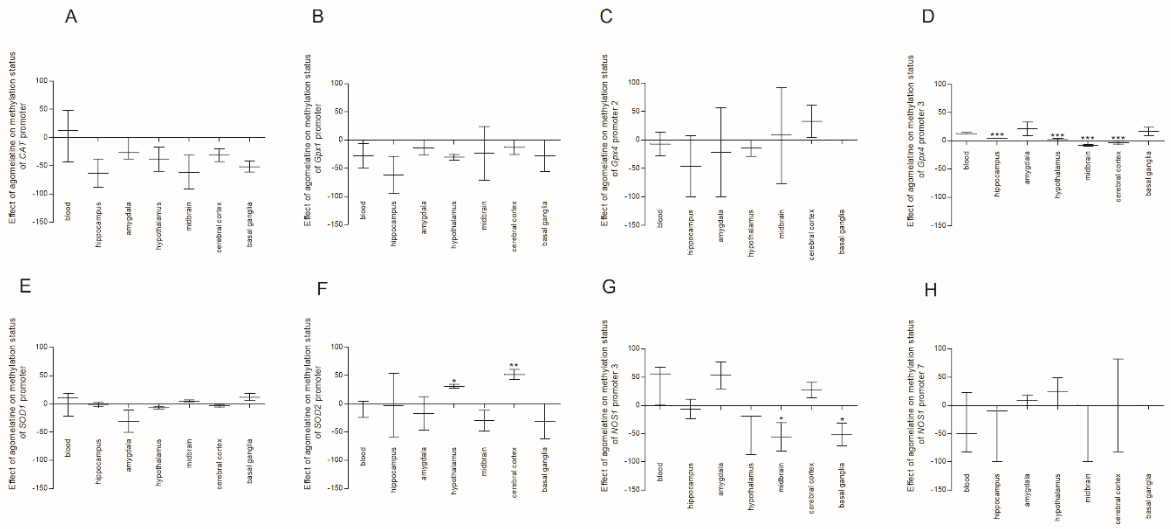

Figure S4. Differences in the methylation status of *CAT* (A), *Gpx1* (B) *Gpx4* promoter 2 (C) promoter 3 (D), *SOD1* (E), *SOD2* (F) and *NOS1* promoter 3 (G), promoter 7 (H) between hippocampus, amygdala, hypothalamus, midbrain, cortex and basal ganglia and PBMCs of animals exposed to CMS procedure for two weeks (Control, Stressed) and in animals exposed to CMS procedure for seven weeks and administered vehicle (1 ml/kg) or agomelatine (10 mg/kg) for five weeks (Control/Ago, Stressed/Saline, Stressed/Ago). Data presented as means ± SEM. N = 6. * $p < 0.05$, ** $p < 0.01$, *** $p < 0.001$ for differences between blood and all studied brain structures.

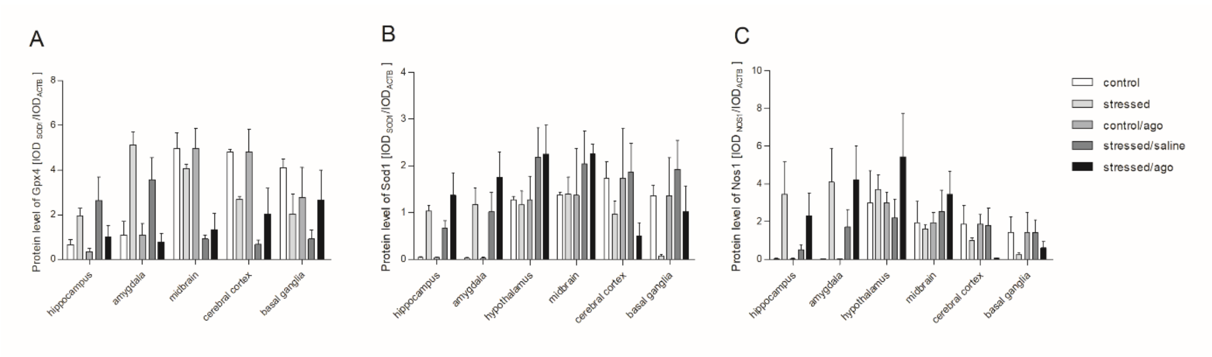

Figure S5. SOD1 (A) and NOS1 (B) protein expression in animals exposed to CMS procedure for two weeks (Control, Stressed) and in animals exposed to CMS for seven weeks and administered vehicle (1 ml/kg) or agomelatine (10 mg/kg) for five weeks (Control/Ago, Stressed/Saline, Stressed/Ago). The graphs show the mean IODs of the bands from all analysed samples. The relative protein expression levels was calculated using the IOD_{gene}/IOD_{ACTB} method . Data presented as means ± SEM. N = 6 N = 6. No significant changes were found between any groups.
